# Salvage chemotherapy in the intensive care unit: a case report of successful treatment of a critically ill patient with metastatic testicular germ cell tumor

**DOI:** 10.3389/fmed.2025.1599413

**Published:** 2025-06-25

**Authors:** Karol Grela, Mykola Sobchynskyi, Mateusz Piȩtak, Barbara Kruczyk, Piotr Domański, Jakub Kucharz, Małgorzata Symonides, Tomasz Demkow

**Affiliations:** ^1^Department of Genitourinary Oncology, Maria Skłodowska-Curie National Research Institute of Oncology, Warsaw, Poland; ^2^Clinical Department of Anesthesiology and Intensive Care, Maria Skłodowska-Curie National Research Institute of Oncology, Warsaw, Poland

**Keywords:** testicular cancer, germ cell tumor, intensive care unit, chemotherapy, dialysis

## Abstract

Testicular cancer, predominant among young males, poses a significant healthcare challenge with varying incidence rates across European regions. Germ cell tumors (GCTs), constituting 95% of the cases, can be divided into seminoma and non-seminomatous germ cell tumors (NSGCTs). Metastases commonly occur in the lungs, lymph nodes, liver, bone, and brain. This study focuses on a 23-year-old with metastatic NSGCT, who underwent chemotherapy in an intensive care unit setting, followed by a remarkable improvement in his condition. The patient’s initial complaint was persistent abdominal pain, which led to a discovery of an extensive tumor in the abdominal cavity. He underwent cytoreductive surgery, and required admission to an intensive care unit (ICU) due to surgical complications. Histopathology identified a yolk sac tumor. Despite the serious condition, chemotherapy was started. The patient’s condition continued to deteriorate, requiring the initiation of mechanical ventilation. However, the multidisciplinary team opted for a continued treatment. Eventually, the AFP levels started to decrease and a gradual improvement of the patient’s condition have been observed. This allowed to a bleomycin, etoposide, and platinum regimen. Following a complex hospitalization and subsequent surgical resection of residual lesions, the patient remains in good clinical condition. Regular follow-up evaluations have revealed no evidence of disease recurrence. Although NSGCT is characterized by a rather favorable prognosis, it is a significant challenge to clinicians, as the diagnosis is often delayed due to the lack or low specificity of the symptoms. Despite the usually young age of the patients diagnosed with metastatic NSGCT, the patients condition can deteriorate rapidly, presenting with multiorgan failure and a need for an ICU treatment. However, the severity of the patients’ condition should not be unequivocally associated with being not suitable for a systemic curative treatment. Such patients can still benefit from the chemotherapy, and a cooperation between the oncologists and critical care physicians is crucial to improving the outcomes and increase the probability of recovery.

## Introduction

Testicular cancer is the most common malignancy affecting young males aged 15–40. Europe bears the highest burden of this disease globally, with varying incidence rates across its regions: Western Europe has an age-standardized rate (ASR) of 8.7 per 100,000 men, Northern Europe follows at ASR 7.2, while Southern and Central Europe has an ASR of 5.9, and Eastern Europe reports ASR of 3.2. Notably, the countries with the highest national ASRs worldwide–Norway, Slovenia, and Denmark–are all located in Europe. In fact, all of the top ten countries with the highest incidence of testicular cancer are European nations (1).

Germ cell tumors (GCTs) account for 95% of testicular cancer cases. For clinical purposes, these tumors are categorized into two principal groups: seminoma and non-seminomatous germ cell tumors (NSGCTs). The incidence ratio between seminoma and NSGCT stands at approximately 1:1.5 (2). It is pertinent to note that GCTs can manifest outside the testicles and in instances where there is no identifiable primary site within the testes, the tumor is classified as extragonadal (3). Lungs, distant lymph nodes, liver, bone, and brain are the most common sites for metastases (4). The majority of cases (64%) are diagnosed at stage I (5); however, due to the rapid growth rate of the tumor, a subset of patients is diagnosed at a metastatic stage with impaired performance status. However, it’s essential to note that even individuals with an extremely advanced disease stage may still have a chance of recovery. Early detection and prompt intervention remain critical in improving outcomes. Diagnostic assessment of these neoplasms relies significantly on imaging studies and specific biomarkers. The primary therapeutic approach for NSGCT involves orchidectomy, followed by chemotherapy.

This study centers on a 23-year-old patient who presented with severely metastatic NSGCT upon admission. Due to his poor general condition, cardiopulmonary insufficiency, and renal failure, the patient required intensive care and was administered systemic treatment in the ICU setting.

## Case description

A 22-year-old male sought medical attention at Proszowice Hospital in October 2019 due to a month-long episode of persistent abdominal pain. An initial ultrasonography revealed a solid lesion on his left flank. A CT scan of the abdomen performed at Proszowice Hospital 1 month later revealed an extensive, irregular tumor measuring 105 × 228 × 206 mm located in the abdominal cavity, accompanied by clusters of para-aortic lymph nodes. A Fine Needle Aspiration Biopsy identified non-epithelial tumor cells, likely of sarcomatoid origin. A scrotal ultrasound detected small calcifications and cystic lesions in the left testicle. Initially, a sarcoma was suspected, leading to cytoreductive surgery at the University Hospital in Krakow on November 27. A non-radical tumor resection was performed, and samples of tumor and omental metastases were collected for histopathological examination. The patient was left with an open abdomen and transferred to the intensive care unit (ICU) due to an unstable condition after surgery, where he required mechanical ventilation (MV), vasopressor therapy, empiric antimicrobial treatment, and blood transfusion. A relaparotomy performed on November 28 resulted in an improvement in the patient’s condition. The patient was discharged from ICU on December 2nd and was then referred to a surgical department. On December 9 he was discharged home. Subsequent histopathology results showed fragments of the tumor exhibited a solid architecture, partially with follicular structures, and in some areas, a looser myxoid stroma. Focally, the tumor formed small glandular structures. The neoplastic cells were predominantly epithelioid, with some areas displaying sarcomatoid features. The cells showed expression of CKAE1/E3 and SALL4, with few expressing Glypican-3. There was no expression of CD30, AFP, or CD117. Findings were suggestive of a malignant germ cell tumor—specifically, a yolk sac tumor.

On December 18, a follow-up CT scan performed at the University Hospital in Krakow revealed tumor progression (the exact size of the lesion was difficult to assess due to the post-surgical status and hemorrhage within the tumor), along with hepatic and peritoneal metastases, bilateral ureteral obstruction due to compression, and a tumor thrombus in the inferior vena cava (IVC). Such an exceptionally rapid progression (tumor recurrence at the resection site, with further progression of lesions resulting in ureteral compression, the presence of liver and peritoneal metastases, as well as tumor mass in the IVC) was indicative of the highly aggressive nature of this malignancy. Following an oncology consultation 5 days later, tumor-specific markers were measured: HCG was 4.95 mlIU/ml within the normal range (<5), AFP concentration was 15,598.25 IU/ml (0.0–5.8), and LDH levels were not tested. Additionally, laboratory tests showed leukocytosis with neutrophilia, anemia (grade 2), thrombocytosis, hyponatremia 121 mmol/l (136.0–146.0), hyperkalemia 6.12 mmol/l (3.50–5.10), a creatinine level of 10.69 mg/dl (0.67–1.17) with eGFR 6.39 ml/min, and a urea level of 35.49 mmol/l (17.0–43.0). Consequently, the patient was diagnosed with acute kidney injury (AKI) and underwent four dialysis sessions and received eight red blood cells transfusions during a 4-day hospitalization at the nephrology department in L. Rydygier Specialist Hospital in Krakow.

By December 27, the patient was referred to the Department of Genitourinary oncology of The Maria Sklodowska-Curie National Research Institute of Oncology in Warsaw and his general condition had deteriorated significantly, presenting cachexia and resting dyspnea. Physical examination findings included tachycardia, bilaterally quiet breath sounds, a palpable abdominal mass, poorly audible bowel sounds, dilated thoracic and abdominal veins, edema in the lower extremities, and slightly drying mucosa. It was decided that in order to safely start salvage chemotherapy, the patient, due to his severe condition, needed to be transferred to the intensive care unit. Moreover, it was decided to administer Carboplatin (CBDCA) with an adjusted dosage of 200 mg/m2 as a prophase in accordance with the experience of our center. Carboplatin was chosen over a short etoposide and cisplatin (EP) regimen due to its more favorable toxicity profile for the patient–the EP regimen was not feasible due to liver injury, defined as an increase in liver enzyme activities: ALT 385 U/L (<50), AST 1693 U/L (<50), LDH 5904 IU/l (<247); without cholestatic pattern: ALP 136 IU/l (30–120), GGTP 40 IU/l (<55). Furthermore, the pharmacokinetic profile of carboplatin allows for more precise dosing during continuous veno-venous hemofiltration (CVVHF). Norepinephrine continuous infusion was administered due to hypotension. Empiric antibiotic therapy was initiated due to possibility of underlying infection.

**FIGURE 1:**
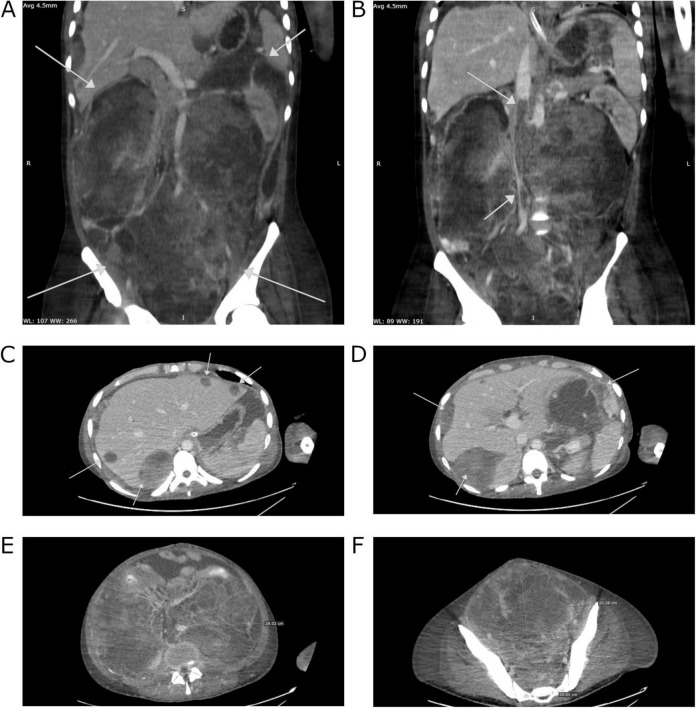
Computed tomography images of the patient’s abdomen from 31.12.2019 performed at The Maria Sklodowska-Curie National Research Institute of Oncology in Warsaw. **(A)** A mass occupying the entire abdominal and pelvic cavity. **(B)** Liver metastases. **(C)** Peritoneal metastases. **(D)** Thrombosis in the inferior vena cava (IVC). **(E)** Tumor mass in cross-section at the level of the mid-abdomen. **(F)** Cross-section at the level of the pelvis.

Over the next few days, the patient’s condition continued to worsen, necessitating supplemental oxygen therapy with a face mask, and later invasive mechanical ventilation. Liver enzymes activity and inflammatory parameters increased, dialysis was upheld. AFP concentrations revealed a significant decrease in comparison to the previous measurements: 3 days after chemotherapy induction – 22,483 IU/ml, 7 days after–21,064 IU/ml, and 10 days after–13,937 IU/ml. The CT scan performed on December 31 showed further tumor progression (258 × 185 × 360 mm) and merged enlarged lymph node masses located retroperitoneally, numerous metastatic lesions in the liver (the largest, located in segment VII, measuring 67 × 57 mm) and in the splenic hilum (47 × 26 mm), multiple peritoneal metastases, and ascites. Additionally, there was thrombosis in the IVC (Figure 1). Despite the patient’s grave condition, a multidisciplinary team opted to continue treatment due to the marked decrease in AFP concentration, administering a second dose of CBDCA (400 mg) in accordance with the ESMO Guidelines and experience of our center (6).

**FIGURE 2:**
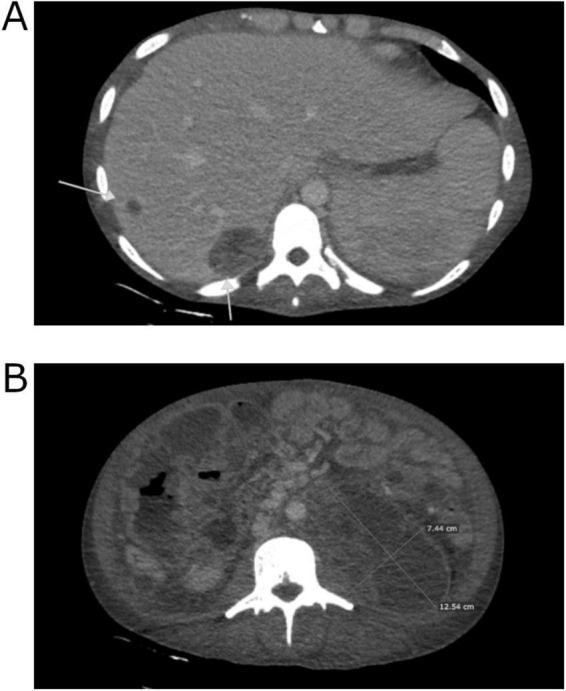
Computed tomography images of the patient’s abdomen from 28.01.2020 performed at The Maria Sklodowska-Curie National Research Institute of Oncology in Warsaw. **(A)** Regression of liver metastases. **(B)** Reduction in tumor size.

In the following weeks, the patient’s condition gradually improved, leading to the withdrawal of invasive ventilation, dialysis, and norepinephrine infusion. A follow-up CT scan indicated significant regression of the tumor (although, according to the reporting radiologist, the change is non-measurable and therefore not suitable for formal assessment according to RECIST criteria). Metastases in the liver and peritoneum were noted, with the largest liver lesion measuring 43 × 32 mm (previously 67 × 51 mm), which meets the criteria for partial response as defined by RECIST guidelines. Confluent lymph node masses and the lesion in the splenic hilum also showed partial regression. Additionally, there was partial recanalization of the IVC (Figure 2). These findings prompted the patient’s transfer to our department. Following additional team consultations, it was decided to initiate radical chemotherapy with a bleomycin, etoposide and platinum (BEP) regimen (four cycles) according to European Society for Medical Oncology (ESMO) Consensus Conference Guidelines on testicular germ cell cancer (2018) (6), with modifications tailored to the patient’s condition (Table 1). For the first 3 days of the cycle, bleomycin was withheld due to ongoing oxygen supplementation. Due to an elevated total bilirubin concentration of 87.4 μmol/L (reference range: 5.0–21.0), the dose of etoposide was reduced by 40% in the first cycle. In the second cycle, because of persistently elevated bilirubin concentration at 32.7 μmol/L, etoposide was administered at 75% of the recommended dose. Considering risk factors, primary prophylaxis of neutropenic fever with filgrastim was implemented. The patient tolerated chemotherapy well, and further decreases in AFP concentration were observed: 2195.0 IU/ml 1 month after induction, 242.6 IU/ml 2 months after induction, and 22.8 IU/ml 3 months after induction. Simultaneously, supportive treatment, nutritional therapy, rehabilitation, and clinical psychology support were provided.

**TABLE 1:** Chemotherapy regimen including cycles, drugs, and dosages.

Cycle	Cycle day	Drug	Dose
CBDCA prophase	1	Carboplatine	400 mg
	17	Carboplatine	400 mg
BEP 1	1	Cisplatine	37 mg
		Etoposide	187 mg
	2	Cisplatine	37 mg
		Etoposide	187 mg
	3	Cisplatine	37 mg
	4	Cisplatine	37 mg
		Bleomycin	30 u
	5	Cisplatine	37 mg
	8	Bleomycin	30 u
	15	Bleomycin	30 u
BEP 2	1	Cisplatine	35 mg
		Etoposide	120 mg
		Bleomycin	30 u
	2	Cisplatin	35 mg
		Etoposide	120 mg
	3	Cisplatine	35 mg
		Etoposide	120 mg
	4	Cisplatine	30 mg
		Etoposide	120 mg
	5	Cisplatine	30 mg
		Etoposide	120 mg
	10	Bleomycin	30 u
	15	Bleomycin	30 u
BEP 3	1	Cisplatine	35 mg
		Etoposide	170 mg
		Bleomycin	30 u
	2	Cisplatine	35 mg
		Etoposide	160 mg
	3	Cisplatine	35 mg
		Etoposide	160 mg
	4	Cisplatine	30 mg
		Etoposide	160 mg
	5	Cisplatine	30 mg
		Etoposide	160 mg
	8	Bleomycin	30 u
	15	Bleomycin	30 u
BEP 4	1	Cisplatine	35 mg
		Etoposide	165 mg
		Bleomycin	30 u
	2	Cisplatine	35 mg
		Etoposide	165 mg
	3	Cisplatine	35 mg
		Etoposide	165 mg
	4	Cisplatine	30 mg
		Etoposide	165 mg
	5	Cisplatine	30 mg
		Etoposide	165 mg
	8	Bleomycin	30 u
	15	Bleomycin	30 u

**FIGURE 3:**
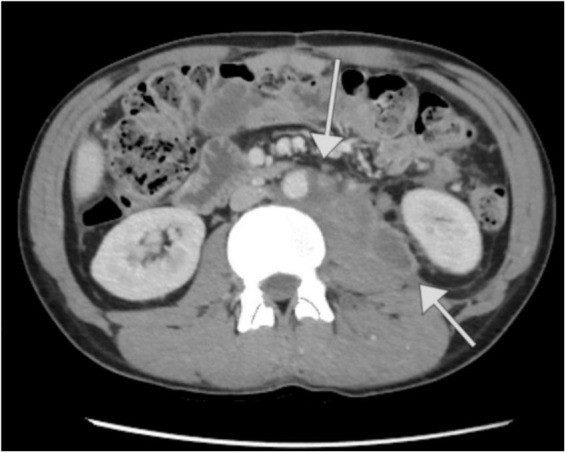
Computed tomography image of the patient’s abdomen after completion of chemotherapy performed at The Maria Sklodowska-Curie National Research Institute of Oncology in Warsaw. Partial tumor regression.

On March 31, after 3 months of hospitalization, the patient was discharged in good general condition, with a performance status assessed as ECOG 2. He attended a follow-up appointment on June 9, reporting a satisfactory state and a weight gain of 18 kg. Laboratory tests showed tumor markers levels within reference values, while a CT scan revealed a partial response to chemotherapy: partial regression of lesions in the liver (e.g., the lesion in segment VII measuring 24 × 16 mm), with smaller lesions showing complete regression. The abdominal tumor demonstrated significant regression, now presenting as several separate intraperitoneal and retroperitoneal lesions with partial necrosis (the largest being periaortal on the left side, measuring 72 × 31 mm). Additionally, there was a reduction in diffuse peritoneal infiltration, partial regression of lymph node clusters, and absence of peritoneal effusions and urinary retention, along with partial recanalization of the inferior vena cava (IVC) compared to the previous scan (Figure 3). The patient’s performance status was reassessed as ECOG 1. Subsequently, he was referred to the Department of Urology in University Hospital in Kraków to explore the potential for retroperitoneal lymph node dissection (RNLD) or multiorgan resection.

**FIGURE 4:**
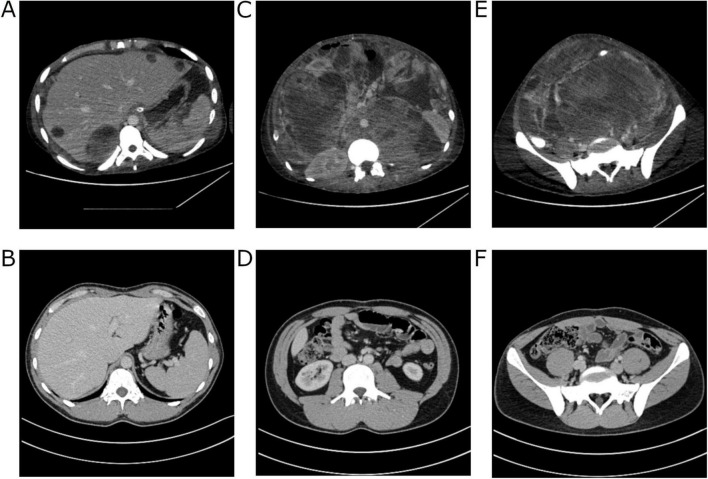
Computed tomography images of the patient’s abdomen performed at The Maria Sklodowska-Curie National Research Institute of Oncology in Warsaw. Comparison of the scan from 31.12.19 with the scan from 08.04.24. Regression of changes in the liver and peritoneal cavity **(A,B)**. Cross-section at the level of the mid-abdomen **(C,D)** showing regression of the primary lesion and notable muscle tissue regeneration. Regression of changes in the pelvis **(E,F)**.

During the surgical intervention, retroperitoneal lymph nodes, along with numerous metastases in the intestines, peritoneum, and liver, were excised, with histopathological examinations revealing only necrosis. Following the surgery, the patient was transferred to the ICU for postoperative management. A total of 5 units of red blood cells were administered. In the ICU, vital functions were continuously monitored, pain management was maintained, and broad-spectrum antibiotic therapy was administered. After 4 days in the ICU, the patient, in stable condition, was transferred to the urology department, and 5 days later he was discharged home, in good general condition. CT performed after hospitalization showed residual lesions without any signs of progression (Figure 4). Over the ensuing 3 years, the patient underwent routine follow-up assessments. In the first year after the completion of treatment, the patient attended follow-up visits every 3 months. During each visit, tumor markers were assessed, and CT scans were performed. In subsequent years, follow-up visits occurred every 6 months, during which tumor marker levels were also measured, and imaging studies (CT or ultrasound) were conducted. Subsequent CT scans exhibited a decrease in lesion size and a sustained stable disease profile (Supplementary Figure 1). The patient maintained normal daily functioning, with no discernible adverse effects from the treatment. The most recent follow-up appointment was scheduled on the 27th of August 2024. The patient attended the visit in good general condition. The CT scan performed on the 8th of April 2024 revealed no signs of recurrence of the disease. Timeline 1 shows the key milestones of diagnosis, treatment, and follow-up in the patient’s clinical course.

## Discussion

Testicular cancer represents a significant healthcare concern, particularly among young men. This neoplasm exhibits a high degree of sensitivity to chemotherapy and often displays a favorable response to treatment. Remarkably, even in cases classified as poor-risk, the 5-year survival rate remains relatively high.

Inguinal orchidectomy remains the first-line treatment for both seminoma and NSGCT. Further management depends on risk stratification, based on factors such as mediastinal primary site, elevated tumor markers (AFP, HCG, LDH), and non-pulmonary visceral metastases (7). These criteria guide chemotherapy selection and prognosis. In poor-risk NSGCT, the 5-year survival is approximately 71%, and four cycles of BEP (bleomycin, etoposide, and carboplatin) are typically recommended (8–10).

One of the main challenges in treating non-seminomatous germ cell tumors (NSGCT) is their often delayed diagnosis. In some cases, patients are first diagnosed when presenting with critical conditions such as renal failure, highlighting the essential role of ICU care. Chemotherapy, while necessary, carries risks of severe complications like tumor lysis syndrome and neutropenic fever, which are better anticipated and managed in the ICU. Moreover, chemotherapy remains a feasible option for patients on dialysis, with established guidelines to support its administration in such cases (11, 12).

The use of chemotherapy in the ICU for patients with hematologic malignancies and multiorgan failure is well-documented (13). Administering chemotherapy to critically ill cancer patients is a complex decision due to uncertain outcomes and limited studies assessing its benefits (14–17). Darmon et al. (16) and Song et al. (15) found that survival after ICU chemotherapy strongly correlates with the number of failing organs, with respiratory and cardiovascular failure being linked to higher mortality. Importantly, ICU mortality was not associated with disease status, including cancer extent or organ involvement, highlighting the urgency of treatment regardless of malignancy stage. These studies support the feasibility and potential benefit of chemotherapy in selected critically ill patients receiving advanced life support. Moreover, Song et al. emphasize that infection, with or without neutropenia, should not be a contraindication, stating that “given the notable sensitivity of certain solid tumors to chemotherapy, it is reasonable to posit that this treatment modality can be efficacious in their therapy as well,” a claim supported by several publications (14, 16, 17).

Another important consideration in the management of patients with advanced testicular cancer is the adjustment of the initial chemotherapy dose. Gillessen et al. compared a retrospective cohort of patients receiving a modified low-dose BOP regimen (babyBOP) with a control group treated with standard BOP. The analysis revealed no statistically significant differences in progression-free survival (PFS) or overall survival (OS) between the two groups (18). On the other hand, Massard et al. demonstrated that in patients with poor-risk NSGCT at high risk of chemotherapy-induced acute respiratory distress syndrome (ARDS), the use of a reduced induction EP regimen was associated with a significantly lower incidence of pulmonary complications compared to those receiving the standard EP protocol (19). In a related study, Tryakin et al. evaluated historical cohorts of patients with metastatic NSGCT and either high tumor burden or poor performance status. Their findings indicated that reducing the dose of the first cycle of EP chemotherapy significantly decreased the rate of life-threatening complications, but did not impact overall survival (20).

These findings collectively underscore the importance of individualized treatment strategies in NSGCT, especially in critically ill patients, where modified regimens and ICU support may improve outcomes without diminishing therapeutic efficacy (21).

In the case described here, success was achieved through the efforts of a multidisciplinary team, which included anesthesiologists responsible for life support and for management of treatment complications, and oncologists responsible for administering chemotherapy that was effective and as safe as possible. Such cooperation allowed to implement treatment with carboplatin as the prephase. The choice of the drug was made due to its acceptable toxicity profile, predictable pharmacokinetics and clear dosing during hemodialysis. According to ESMO guidelines, carboplatin is recommended over cisplatin in patients with cancer-related renal impairment, if renal function improvement is believed to be possible (6). Concurrently, an intensive life support and management of multiple organ failure was possible. Treating an oncology patient in the ICU presents challenges arising from the necessity of continuous communication between anesthesiologists and oncologists, with potential difficulties in understanding the capabilities and limitations of various procedures. Simultaneously, ICU treatment allows for the rapid correction of disturbances and complications resulting from both the disease and the administered treatments. We believe that the simultaneous administration of salvage chemotherapy and intensive care provided in the ICU, despite the patient’s critical condition, played a crucial role in saving the patient’s life. The choice of carboplatin with adjusted dosage proved to be both safe and effective in this patient. The prompt initiation of chemotherapy on the day of admission prevented a further significant deterioration in the patient’s condition. Treatment of shock and respiratory failure, as well as renal replacement therapy, served as a bridge to stabilize the patient, enabling the subsequent application of definitive treatment.

## Conclusion

Patients with advanced cancer are typically perceived as ineligible for intensive treatments. However, as exemplified by this case, testicular tumors have demonstrated the potential for complete curability even in such advanced stages. This underscores the importance of diligent clinical assessment and the consideration of treatment options in severe cases, ultimately aiming to improve outcomes and offer hope for recovery.

## Data Availability

The original contributions presented in this study are included in this article/Supplementary material, further inquiries can be directed to the corresponding author.

## Supplementary material

Supplementary Figure 1 This timeline outlines the key stages of diagnosis and treatment.
