# Supplementary figures and images for: Salvage chemotherapy in the intensive care unit: a case report of successful treatment of a critically ill patient with metastatic testicular germ cell tumor
Source: Front Med (Lausanne). 2025 Jun 25;12:1599413. doi: 10.3389/fmed.2025.1599413 (PMC12237988; doi:10.3389/fmed.2025.1599413)

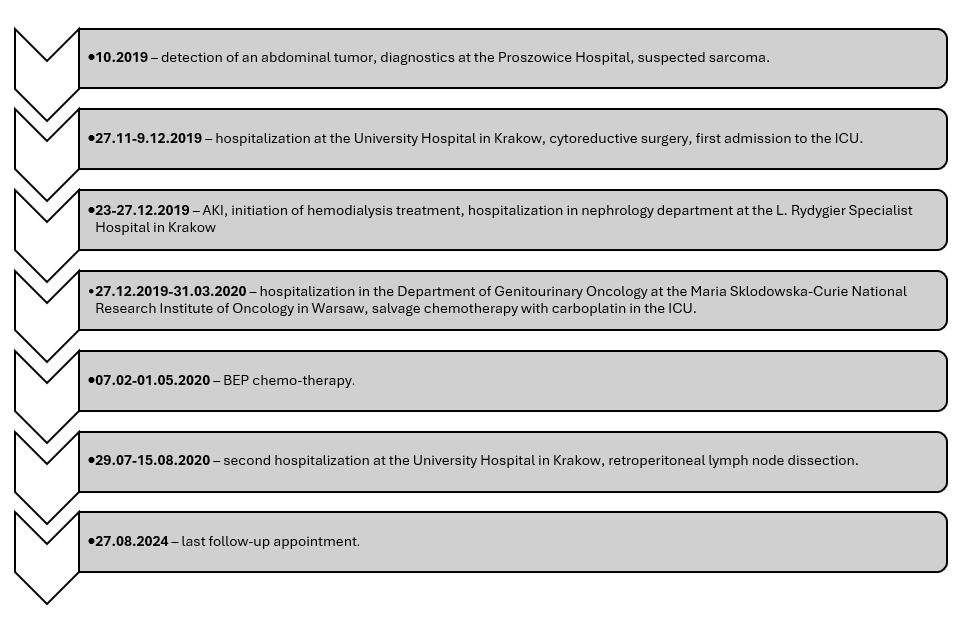

Supplement: Supplementary Figure 1 — This timeline outlines the key stages of diagnosis and treatment. [file Image_1.tif]
